# President’s Address before the California State Homœopathic Society

**Published:** 1883-08

**Authors:** 


PRESIDENT'S ADDRESS before the California State Homœopathic Society. By J. M. Selfridge, M. D.

An interesting and able address. One remark we would quote and indorse: "Another duty in the cause is to teach our medical students pure Homœopathy. To this end we should discourage their attendance upon *any* school of medicine that does not teach the system in its purity." * * * "The fac is apparent that in every school of medicine there are too many colleges."

Homœopathy, especially, has too many colleges pretending to teach it. Some are good; others *very* poor.

